# Development of an optimized method for the detection of airborne viruses with real-time PCR analysis

**DOI:** 10.1186/1743-422X-8-369

**Published:** 2011-07-27

**Authors:** Panos G Ziros, Petros A Kokkinos, Euaggelia Legaki, Apostolos Vantarakis

**Affiliations:** 1 Environmental Microbiology Unit, Department of Public Health, School of Medicine, University of Patras, Rion, GR 26504, Greece

**Keywords:** airborne viruses, air sampling, air pollution, human adenovirus, norovirus, and wastewater treatment plant

## Abstract

**Background:**

Airborne viruses remain one of the major public health issues worldwide. Detection and quantification of airborne viruses is essential in order to provide information regarding public health risk assessment.

**Findings:**

In this study, an optimized new, simple, low cost method for sampling of airborne viruses using Low Melting Agarose (LMA) plates and a conventional microbial air sampling device has been developed. The use of LMA plates permits the direct nucleic acids extraction of the captured viruses without the need of any preliminary elution step. Molecular detection and quantification of airborne viruses is performed using real-time quantitative (RT-)PCR (Q(RT-)PCR) technique. The method has been tested using Adenoviruses (AdVs) and Noroviruses (NoVs) GII, as representative DNA and RNA viruses, respectively. Moreover, the method has been tested successfully in outdoor experiments, by detecting and quantifying human adenoviruses (HAdVs) in the airborne environment of a wastewater treatment plant.

**Conclusions:**

The great advantage of LMA is that nucleic acids extraction is performed directly on the LMA plates, while the eluted nucleic acids are totally free of inhibitory substances. Coupled with QPCR the whole procedure can be completed in less than three (3) hours.

## Background

Viruses are pathogenic to humans and animals. There is a growing concern regarding exposure to bioaerosols since they represent major health and economic risks to human and animal populations. Exposure to airborne viruses in different environments is responsible for various health problems and disorders worldwide [1]. Moreover, there is an increased concern of using highly pathogenic airborne microorganisms, as bioterrorism agents. Aerosolization of viral pathogens occurs in processes such as spray irrigation of wastewater and operation of sewage treatment plants, but also from humans and animals as a result of coughing, sneezing, breathing, especially in indoor air environments [1-3]. The question of airborne transmission is especially important for the waste treatment plant workers and for healthcare workers where patients tend to congregate during for example influenza season [4]. Many highly pathogenic viruses, such as SARS coronavirus (SARS-CoV), which causes severe acute respiratory syndrome, a highly infectious disease with a significant mortality, have the potential of being converted from droplet to airborne transmission and for that reason health care workers are particularly vulnerable [5]. Airborne spread of the SARS epidemic has been suggested for the transmission of the disease in Hong Kong in 2003 and several epidemiological studies have proposed an airborne transmission for various pathogenic viruses [6-10].

To control and prevent exposure to airborne viruses, efficient monitoring through accurate sampling is fundamental. Many types of samplers have been used over the years, including liquid impingers, solid impactors, filters, electrostatic precipitators, and many others [8]. In this study, we used low melting agarose (LMA) as a capture medium instead of agar based media, since preliminary spiking experiments using Tryptic Soy Agar (TSA) resulted in very pure recovery efficiencies (5-10% recoveries, data not shown), due to inhibition of the final Q(RT-)PCRs. Agar itself has been reported to cause inhibition of the PCR assay [11]. On the other hand, the use of LMA has been shown to improve the PCR amplification from templates which were naturally contaminated by various PCR inhibitors [12].

## Methods

### Preparation of LMA plates and air sampling

Airborne viruses were captured on LMA plates (0.5% LMA) in acidic conditions (0.05 M Glycine, pH 3.5). LMA plates were prepared by dissolving 0.5 gr of Low Melting Agarose (Invitrogen) in 100 ml of Glycine buffer, pH 3.5. The LMA solution was autoclaved for 20 min and after being cooled down to 40°C in a water bath, (2 ml) was transferred to 60 mm Petri dishes. The LMA plates were left at room temperature in order to solidify and stored at 4°C until used. Air was sampled at a programmable flow rate using the Surface Air System (SAS International Pbi, Italy), an impactor sampler with contact agar plates. These plates were filled with 2 ml of LMA instead of agar, as described above.

### Spiking experiments

Spiking experiments were performed using Human Adenovirus 35 (HAdV-35) and Human Norovirus GII (NoV) (kindly donated by Annika Allard, Umeå University, Sweden). Virus titres of approximately 10^5 ^copies/ml for HAdV, and 10^6^copies/ml for NoV were used. In order to test the efficiency of virus recovery from LMA plates, three (3) different sets of samples were spiked with serial dilutions of 100 μl viral suspensions in PBS and spread on the surface of the plates, using a 2 μl eppendorf pipette. LMA plates were seeded with (i) HAdV containing 1 × 10^4 ^GC (ii) HAdV containing 1 × 10^3 ^GC (iii) HAdV containing 1 × 10^2 ^GC (Table 1). Before NA extraction, all samples were placed on SAS air sampler and air sampling was performed under sterile conditions for 20 min (at constant flow rate of 150 lt per minute) in a level two cell culture laminar flow. NA extraction was performed directly from the LMA plates and virus recovery was estimated with QPCR for HAdV. In a second set of spiking experiments, an RNA virus was tested. To this end, NoV GII was used in three different sets of samples spiked with 1 × 10^4^, 1 × 10^3 ^and 1 × 10^2 ^GC of NoV, as shown in Table 2. NA extraction was performed directly from the LMA plates and virus recovery was estimated with QRT-PCR for NoV GII.

**Table 1:** Recovery values of HAdV 35 seeded in LMA plates

Sample ID	HAdV added GC/plate	HAdV recovered GC/plate	Recovery (%)	Mean recovery (%)
exp1	1 × 10^4^	0,47 × 10^4^	47	81
exp2	1 × 10^4^	1,01 × 10^4^	101	
exp3	1 × 10^4^	0,95 × 10^4^	95	
exp4	1 × 10^3^	0,59 × 10^3^	59	60,3
exp5	1 × 10^3^	0,59 × 10^3^	59	
exp6	1 × 10^3^	0,6 × 10^3^	63	
exp7	1 × 10^2^	0,42 × 10^2^	42	33,7
exp8	1 × 10^2^	0,26 × 10^2^	26	
exp9	1 × 10^2^	0,33 × 10^2^	33	

**Table 2:** Recovery values of NoV GII seeded in LMA plates

Sample ID	NoV GII added GC/plate	NoV GII recovered GC/plate	Recovery (%)	Mean recovery (%)
exp1	1 × 10^4^	0,94 × 10^4^	93	90,3
exp2	1 × 10^4^	1,06 × 10^4^	106	
exp3	1 × 10^4^	0,72 × 10^4^	72	
exp4	1 × 10^3^	0,91 × 10^3^	91	93,3
exp5	1 × 10^3^	1,07 × 10^3^	107	
exp6	1 × 10^3^	0,82 × 10^3^	82	
exp7	1 × 10^2^	0,93 × 10^2^	93	63,3
exp8	1 × 10^2^	0,68 × 10^2^	68	
exp9	1 × 10^2^	0,29 × 10^2^	29	

### Nucleic acid extraction

Nucleic acid (NA) extraction from LMA plates was performed using the NucliSENS Magnetic extraction Reagents (bioMerieux, France) according to kit instructions, with the following minor modifications. In detail, 4 ml of NucliSENS lysis buffer were added to each LMA plate. Upon addition of lysis buffer LMA was easily dissolved. Then the lysate was transferred to 15 ml falcon tube, briefly vortexed and incubated for 10 min at 55°C in a water bath. Afterwards, 50 μl of well-mixed magnetic silica were added to the lysate and the instructions of the manufacturer were followed until the final step of NA elution. NAs were finally extracted in 50 μl of elution buffer, and extracts were stored at -20°C until analyzed by QPCR. It is worthy of note that any commercial or homemade nucleic acid extraction reagent using a lysis buffer based on GuSCN can be used.

### Real time Q(RT-)PCR analyses

The quantitative real-time PCR (QPCR) for human Adenovirus based on the method described by Hernroth and colleagues [13], was performed in a final volume of 25 μl, using the TaqMan Universal PCR master mix (Invitrogen). The sensitivity of this QPCR HAdV assay has been estimated to be of 1-10 genome copies [13,14]. The following oligonucleotide primers and conditions were used. Forward primer: AdF (5'- CWT ACA TGC ACA TCK CSG G-3'), at a final concentration of 0.9 μM, Reverse primer: AdR (5'- CRC GGG CRA AYT GCA CCA G-3'), at a final concentration of 0.9 μM, Adenovirus TaqMan Probe: AdP1 (5'- FAM- CCG GGC TCA GGT ACT CCG AGGCGT CCT-BHQ-3'), at a final concentration of 0.225 μM. The temperature-time program was as follows: 2 min at 50°C, 10 min at 95°C as a hot start, and 45 cycles of 15 s at 95°C for denaturation, 1 min at 60°C for denaturation, annealing-extension. Standard curves used in the QPCR were generated by using serial dilutions of purified viral genomic DNA from adenovirus 35 (HAdV-35) viral stocks.

The quantitative real-time RT-PCR (QRT-PCR) for human Norovirus GII (detection limit < 10 copies per reaction) based on a method described previously [15], was performed in a final volume of 20 μl, using the RNA UltraSense™ One-Step Quantitative RT-PCR System (Invitrogen). The following primers and conditions were used. Forward primer: QNIF2 (5'- ATG TTC AGR TGG ATG AGR TTC TCW GA -3'), Reverse primer: COG2R (5'- TCG ACG CCA TCT TCA TTC ACA -3'), Norovirus GG II Probe: QNIFS (5'- FAM- AGC ACG TGG GAG GGC GAT CG -BHQ1-3'). The temperature-time program was as follows: 15 min at 50°C for the RT reaction, 10 min at 95°C as a hot start, and 45 cycles of 15 s at 95°C for denaturation, 1 min at 60°C for denaturation, annealing-extension. Standard curves used in the QRT-PCR were generated by using serial dilutions of purified viral genomic RNA from NoV GII viral stocks.

Internal amplification controls (IAC) for human Adenovirus (YORKBIO Cat# IAC-HADV, UK) and Norovirus GII (YORKBIO Cat# IAC-NVGII, UK) were used in order to test the eluted nucleic acids for the presence of inhibitory substances. The following TaqMan probe was used, IAC probe: IACP (5'-VIC-CCA TAC ACA TAG GTC AGG -MGBNFQ-3'), while the primers and conditions were the same to those for HAdV and NoVGII QPCRs, respectively.

Standard precautions were applied in all Q(RT-)PCR assays, such as use of separate work areas and pipettes for pre- and post-amplification steps.

### Outdoor experiments

In order to test the LMA method of air sampling under field conditions, air sampling experiments were performed in an urban area of the city of Patras, located in south-western Greece, and in the sewage treatment plant of Patras' University (SWPT). Samplings at the SWPT were performed during a two months period of summer 2010 at the plant's Aerobic Wastewater Mechanical Aeration Treatment System. Each air sampling was performed for 20 min, which corresponds to 3000 lt of air at a distance of 1 m from the sewage tank, except for two samples which where collected outside the SWTP facilities, at a distance of approximately 50 m from the sewage tank (Table 3). This SWTP operates periodically every 3 hours for 30 min. Samplings were performed under both conditions, during operation or immediately after the end of an operation cycle (Table 3). After sampling, the LMA plates were processed on the same day for NA extraction. NA extracts (50 μl) were stored at -20 C until analyzed.

**Table 3:** Detection and quantification of Human Adenovirus in the air of SWTP

Sample ID	SWTP operation	Adenovirus load per 3000 lt of air	Distance from the tank (m)
A3559	yes	384	1
A3560	yes	116	1
A3561	no	39	1
B3568	no	6,5	1
B3569	no	ND	1
A3602	yes	75	1
A3603	yes	79	1
A3604	no	6,7	1
C3622	no	69	1
C3624	yes	1017	1
C3623	no	83	1
H3761	yes	34	50
H3762	no	ND	50

Detection and quantification of Human Adenovirus in the air of a SWTP using the LMA method and QPCR. Sample IDs with the same initiating letter were sampled during the same day. (ND: Not detected)

**Figure 1:**
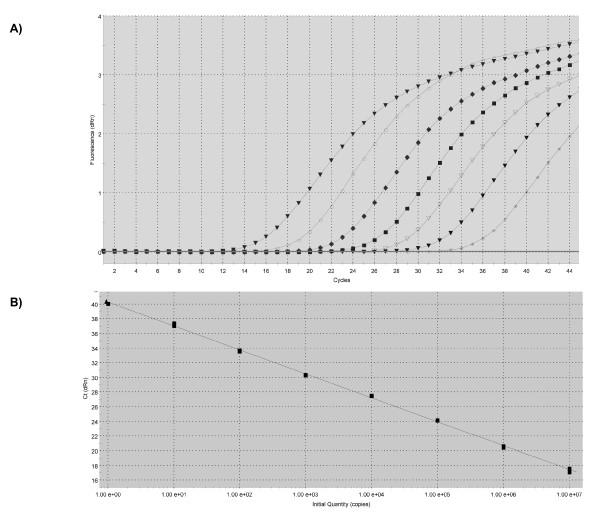
**Real-time -PCR quantification of HAdV 41 standard plasmid**. A) Amplification plots of fluorescence intensities (ΔRn) versus the PCR cycle numbers are displayed for serial 10-fold dilutions of standard plasmids (1 × 10^7 ^to 1 copy per reaction). B) Standard curve generated using a 10-fold dilution series (1 × 10^7 ^to 1 copies per reaction) of HAdV 41 standard plasmid. Slope = -3.262, *Y*-intercept = 40.27, *R*^2 ^= 0.999.

QPCR for HAdV detection and quantification were performed under the same experimental conditions described previously, except for the construction of the standard curve which was generated by using serial dilutions (range 1 to 10^7^) of known amounts of a linearized plasmid containing the entire hexon region of HAdV 41 (kindly donated by Annika Allard, Umeå University, Sweden) (Figure 1).

## Results

### Efficiency of viral recovery from LMA plates

HAdV recovery from LMA plates was estimated with QPCR. Mean values of three independent experiments for the determination of viral recovery were 81% (for the samples spiked with 1 × 10^4 ^GC), 60.3% (for the samples spiked with 1 × 10^4 ^GC), and 33.7% (for the samples spiked with 1 × 10^2 ^GC) (Table 1).

NoV GII recovery from LMA plates was estimated with QRT-PCR. The mean viral recovery values of three independent experiments were 90.3% (for the samples spiked with 1 × 10^4 ^GC), 93.3% (for the samples spiked with 1 × 10^4 ^GC), and 63.3% (for the samples spiked with 1 × 10^2 ^GC) (Table 2).

The NA extracts from the field experiments were tested for inhibition against control samples of pure NA elution buffer, with the use of previously described IACs. No statistically significant difference was observed between the LMA samples and the control samples. Regarding HAdV, the mean Ct value for LMA samples was 34.4 (SD 0.44), while the Control Ct was 34.4 (SD 0.39). For the NoVGII analyses, the mean Ct values for the LMA samples was 30.53 (SD 0.27), while the Control Ct value was 30.29 (SD 0.38).

### Detection of viruses in outdoor experiments

As it is shown in Table 3, HAdV were detected in eleven (11) out of thirteen (13) samples collected at the SWTP. At the Aerobic Wastewater Mechanical Aeration Treatment System the results indicated a considerable airborne viral pollution, with HAdV detected in 84.6% of the tested samples. Interestingly, 100% of the samples were found to be positive when the SWTP was in operation. HAdV were not detected in anyone of the samples (six samples) originating from the urban area of the city. QRT-PCR for Norovirus GII detection were also performed in the same samples. No positive NoV GII samples were detected, neither to the urban area, nor to the wastewater treatment plant.

## Discussion and conclusions

The results of the present study showed that Low Melting Agarose (LMA) can be efficiently used as a capturing medium of airborne viruses in solid impactor air samplers. Usually, sampling with this type of air samplers is performed using agar based media [8]. The drawback of agar plates is that viruses have to be eluted from the agar plates before the subsequent nucleic acid extraction step, which results in viral losses. Moreover, inhibitory substances presented in the agar media tend to remain during the subsequent NA isolation and finally inhibit the Q(RT-)PCR reactions, thus considerably reducing the detection limit of airborne viruses. In contrary, the great advantage of LMA is that NA extraction is performed directly on the LMA plates, while the eluted NA is totally free of inhibitory substances. Coupled with QPCR the whole procedure can be completed in less than three (3) hours.

In accordance to the findings of others studies, which support that the wastewater treatment plants are sources of considerable airborne contamination, which may pollute the environment and constitute an important biological hazard for workers [2,16,17], the present study revealed a significantly high level of viral contamination during the operation of the studied SWTP.

We intend to apply this optimized method for the detection of airborne viruses in indoor and outdoor environments.

## List of abbreviations

HAdV: human adenovirus; NoV: norovirus; LMA: low melting agarose; SWTP: sewage treatment plant; QPCR: quantitative real time PCR; GC: genome copies; NA: nucleic acids.

## Competing interests

The authors declare that they have no competing interests.

## Authors' contributions

PZ was responsible for setting up the study, performed the samplings at the SWTP, and participated in the molecular analyses and in the writing of the manuscript. PK performed the samplings at the urban areas, and participated in the writing and revision of the manuscript; EL performed the nucleic acid extractions, while AV was responsible for coordinating the study, and drafted the manuscript. All authors read and approved the final manuscript.
